# MicroRNA-21 regulates prostaglandin E2 signaling pathway by targeting 15-hydroxyprostaglandin dehydrogenase in tongue squamous cell carcinoma

**DOI:** 10.1186/s12885-016-2716-0

**Published:** 2016-08-25

**Authors:** Qianting He, Zujian Chen, Qian Dong, Leitao Zhang, Dan Chen, Aditi Patel, Ajay Koya, Xianghong Luan, Robert J. Cabay, Yang Dai, Anxun Wang, Xiaofeng Zhou

**Affiliations:** 1 Center for Molecular Biology of Oral Diseases, Department of Periodontics, College of Dentistry, University of Illinois at Chicago, Chicago, IL USA; 2 Department of Oral and Maxillofacial Surgery, the First Affiliated Hospital, Sun Yat-Sen University, Guangzhou, China; 3 Department of Oral and Maxillofacial Surgery, Nan Fang Hospital, Southern Medical University, Guangzhou, China; 4 Department of Oral Biology, College of Dentistry, University of Illinois at Chicago, Chicago, IL USA; 5 Department of Pathology, College of Medicine, University of Illinois at Chicago, Chicago, IL USA; 6 Department of Bioengineering, College of Engineering, University of Illinois at Chicago, Chicago, IL USA; 7 UIC Cancer Center, Graduate College, University of Illinois at Chicago, Chicago, IL USA; 8 Guanghua School and Research Institute of Stomatology, Sun Yat-sen University, Guangzhou, China

**Keywords:** microRNA, microRNA-mRNA regulatory module, miR-21, HPGD, PGE2

## Abstract

**Background:**

Oral tongue squamous cell carcinoma (OTSCC) is one of the most aggressive forms of head and neck/oral cancer (HNOC), and is a complex disease with extensive genetic and epigenetic defects, including microRNA deregulation. Identifying the deregulation of microRNA-mRNA regulatory modules (MRMs) is crucial for understanding the role of microRNA in OTSCC.

**Methods:**

A comprehensive bioinformatics analysis was performed to identify MRMs in HNOC by examining the correlation among differentially expressed microRNA and mRNA profiling datasets and integrating with 12 different sequence-based microRNA target prediction algorithms. Confirmation experiments were performed to further assess the correlation among MRMs using OTSCC patient samples and HNOC cell lines. Functional analyses were performed to validate one of the identified MRMs: miR-21-15-Hydroxyprostaglandin Dehydrogenase (HPGD) regulatory module.

**Results:**

Our bioinformatics analysis revealed 53 MRMs that are deregulated in HNOC. Four high confidence MRMs were further defined by confirmation experiments using OTSCC patient samples and HNOC cell lines, including miR-21-HPGD regulatory module. HPGD is a known anti-tumorigenic effecter, and it regulates the tumorigenic actions of Prostaglandin E2 (PGE2) by converts PGE2 to its biologically inactive metabolite. Ectopic transfection of miR-21 reduced the expression of HPGD in OTSCC cell lines, and the direct targeting of the miR-21 to the HPGD mRNA was confirmed using a luciferase reporter gene assay. The PGE2-mediated upregulation of miR-21 was also confirmed which suggested the existence of a positive feed-forward loop that involves miR-21, HPGD and PGE2 in OTSCC cells that contribute to tumorigenesis.

**Conclusions:**

We identified a number of high-confidence MRMs in OTSCC, including miR-21-HPGD regulatory module, which may play an important role in the miR-21-HPGD-PGE2 feed-forward loop that contributes to tumorigenesis.

**Electronic supplementary material:**

The online version of this article (doi:10.1186/s12885-016-2716-0) contains supplementary material, which is available to authorized users.

## Background

Head and neck/oral cancer (HNOC) is a commonly encountered malignancy. Head and neck squamous cell carcinoma (HNSCC), which arises from the epithelium lining of this region, makes up the majority (over 90 %) of HNOC. Oral tongue squamous cell carcinoma (OTSCC) is one of the most aggressive form of HNSCCs, which exhibits a propensity for rapid local invasion and spread [1], has a distinct nodal metastasis pattern [2, 3]. OTSCC patients also suffer from a high recurrence rate [4]. OTSCC is a complex disease with extensive genetic and epigenetic defects, including microRNA deregulation. MicroRNAs are pivotal regulators of physiological and disease processes through their control of diverse cellular processes. Several microRNAs have been functionally classified as oncogenes or tumor suppressors, and the aberrant expression of microRNA has been observed in almost all cancer types including OTSCC [5–8]. Deregulation of these cancer-associated microRNAs can significantly impact tumor initiation and progression by activating pathways promoting uncontrolled proliferation, favoring survival, inhibiting differentiation, and promoting invasion [9, 10]. MicroRNAs are not directly involved in protein coding, but are able to control the expression of their target genes at post-transcriptional levels by facilitating mRNA degradation and/or repressing translation. As such, the identification and detection of functional microRNA-mRNA regulatory modules (MRMs) are crucial components for understanding of microRNA functions.

MicroRNAs are a class of small non-coding RNAs of approximately 22 nucleotides in length that are endogenously expressed in mammalian cells. They are related to, but distinct from, siRNAs. A key difference between siRNA and microRNA is that siRNA requires almost complete complementary to its targeting sequence for it to exert the silencing function, whereas microRNA usually binds to its target genes through partial complementary. While numerous sequence-based bioinformatics methods for microRNA target prediction have been developed, these methods often lead to high false discovery rates [11]. In order to minimize false positives and to detect the functional microRNA targets under a specific biological condition, recent approaches often integrate the microRNA and mRNA profiling analysis in conjunction with the sequence-based target prediction. Two types of experiments are common: 1) differential mRNA profiling experiment on a microRNA transfected cell line and its negative control, and 2) simultaneous microRNA and mRNA profiling analysis on samples of different phenotypes (e.g., normal vs. tumor). The first approach has been used by many groups, including us, to define the functional microRNA targets when a specific microRNA is over- or under- expressed [12–14]. The second approach aims to discover microRNA with altered expression related to different phenotypes and to uncover their targets mRNAs. This approach is based on the simple principle that inverse relationships in their expression profiles should be held between a specific microRNA and its functional target genes. When integrated with the sequence-based bioinformatics target prediction, this approach is believed to lead to the identification of high confidence microRNA targets.

Our group and several others have recently undertaken extensive RNA-based surveys to identify gene expression and microRNA abnormalities in OTSCC. In this study, we utilized our existing transcription profiling dataset [15], and a meta-analysis of 13 published microRNA profiling studies [16], and integrate them with a collection of 12 sequence-based bioinformatics tools to define the deregulation of functional MRMs in OTSCC. We then evaluated these MRMs in 2 OTSCC patient cohorts and a panel of HNSCC cell lines. With our comprehensive approach, we identified a panel of high confidence microRNA-mRNA regulatory modules in OTSCC, including miR-21-15-Hydroxyprostaglandin Dehydrogenase (HPGD) regulatory module. We also confirmed the positive feed-forward loop that involves miR-21, HPGD and Prostaglandin E2 (PGE2) in HNOC cells that contribute to tumorigenesis.

## Methods

### MicroRNA target prediction

The microRNA target prediction was performed using the comparative analysis function of the miRWalk [17], which contains a collection of 10 bioinformatics tools, including DIANAmT, miRanda, miRDB, miRWalk, RNAhybrid, PicTar (4-way), PicTar (5-way), PITA, RNA22, TargetScan5.1. In addition, MicroCosm 5.0 and TargetScanHuman 6.2 were also used for predicting the microRNA targets. For our study, genes that were predicted by at least one method were defined as candidate microRNA targets. The base-pairing and the minimum free energy (mfe) for the binding of microRNA to its targeting sequences were predicted using the RNAhybrid program [18].

### Cell Culture, transfection and function assays

The human HNSCC cell lines (1386Ln [19], 1386Tu [19], 686Ln [20], 686Tu [20], CAL27 [21], SCC2 [22], SCC4 [22], SCC9 [23], SCC15 [23], SCC25 [23], Tca8113 [24], UM1 [25], UM2 [25]) were maintained in DMEM/F12 medium (Gibco) supplemented with 10% FBS, 100 units/ml penicillin, and 100 μg/ml streptomycin (Invitrogen). All cells were maintained in a humidified incubator containing 5 % CO_2_ at 37 °C. For functional analysis, hsa-miR-21 and non-targeting microRNA mimic (Dharmacon), and gene specific siRNAs for COX2 and HPGD (Santa Cruz Biotechnology) were transfected into the cells using DharmaFECT Transfection Reagent 1 as described previously [26, 27]. For PGE2 treatment, 20 μM of PGE2 or vehicle (DMSO) was added to the cells and incubated for 24 h. For CelecoxiB treatment, 10 μM of CelecoxiB or vehicle (DMSO) was added to the cells and incubated for 24 h. Cell proliferation was measured by MTT assay as described previously [28].

### Clinical samples from OTSCC patients

We downloaded the RNASeq and miRNASeq profiling datasets on 12 OTSCC and paired normal tissue samples from The Cancer Genome Atlas (TCGA) Data Protal [tcga-data.nci.nih.gov]. The gene expression values were extracted as normalized count, and the microRNA levels were extracted as reads per million miRNA mapped from the datasets. The demographics of the patients were as follows: 6 male, 6 female and average age = 62 (range: 36–88), 1 stage T1 cases, 5 stage T2 cases, 3 stage T3 case and 3 T4 cases. Oral cytology samples were obtained from 13 patients with pathologically characterized primary OSCC of the tongue before tumor resection (including 6 stage T1 cases 6 stage T2 cases and 1 stage T3 case) as previously described [29, 30]. These procedures are in compliance with the Helsinki Declaration, and was approved by the Ethical Committee of the First Affiliated Hospital, Sun Yat-Sen University (reference number: 2014-C-001). The informed consent was obtained from participants. Patients were excluded if there is a history of lung carcinoma or HNSCC elsewhere and may represent metastatic disease. The demographics of the patients were as follows: 8 male, 5 female and average age = 51.8 (range: 32–78). The total RNA was isolated using miRNeasy Mini kit (Qiagen), and quantified by a spectrophotometer or the RiboGreen RNA Quantitation Reagent (Molecular Probes).

### Quantitative RT-PCR Analysis

The relative microRNA levels were determined by TaqMan microRNA assays (Applied Biosystems) as previously described [16, 31]. The relative mRNA levels were determined by quantitative two-step RT-PCR assay with pre-designed gene specific primer sets (Origene) as described before [16, 31]. The relative microRNA and mRNA levels were computed using the 2^-delta delta Ct^ analysis method, where U6 and beta-actin were used as internal controls, respectively.

### Western-blot analysis

Western blots were performed as described previously [16] using antibodies specific for HPGD (Cayman Chemical) and beta-actin (Sigma-Aldrich) and an immuno-star HRP substrate Kit (Bio-RAD).

### Fluorescent immunocytochemical analysis

Immunofluorescence analysis was performed as previously described [16]. In brief, cells were cultured on 8 chamber polypropylene vessel tissue culture treated glass slides (Millipore) fixed with cold methanol, permeabilized with 0.5 % Triton X-100/PBS, and blocked with 1% BSA in PBS. The slides were incubated with primary antibodies against HPGD (1:500, Cayman Chemical). The slides were then incubated with a FITC-conjugated anti-rabbit IgG antibody (1:50, Santa Cruz). The slides were mounted with ProLong Gold antifade reagent containing DAPI (Invitrogen) following the manufacturer’s protocol. The slides were then examined with a fluorescence microscope (Carl Zeiss).

### Dual-Luciferase reporter assay

The luciferase reporter gene constructs (pGL-E1 and pGL-E2E3) were created by cloning a 55-bp fragment from the 3′-UTR (position 2625–2680 of the HPGD mRNA sequence NM_000860, containing the miR-21 site E1) and a 61-bp fragment from the 3′-UTR (position 2860–2921 of the HPGD mRNA sequence NM_000860, containing the miR-21 targeting sites E2 and E3) into the Xba I site of the pGL3-Control firefly luciferase reporter vector (Promega) as described previously [9]. The corresponding mutant constructs (pGL-E1m, pGL-E2mE3, pGL-E2E3m and pGL-E2mE3m) were created by replacing the seed regions (positions 2–8) of the miR-21 binding sites with 5′-TTTTTTT-3′. All constructs were verified by sequencing. The reporter constructs and the pRL-TK vector (Promega) were co-transfected using Lipofectamine 2000 (Invitrogen). The luciferase activities were then determined as described previously [26] using a GloMax 20/20 luminometer (Promega). Experiments were performed in quadruplicate.

### Statistical analysis

Data was analyzed using the Statistical Package for Social Science (SPSS), version 17.0. Student’s *t*-test was used to compare differences between groups. Pearson’s correlation coefficient was computed for examining the relationship between the expression of microRNA and their target genes. For all analyses, *p* < 0.05 was considered statistically significant.

## Results

We first developed a list of putative microRNA-mRNA regulatory modules (MRMs) based on the simple principle that inverse relationships should be anticipated in the expression of a specific microRNA and its functional target gene (mRNA). We used a total of 97 differentially expressed coding genes (44 up-regulated and 53 down-regulated mRNAs, see Additional file 1: Table S1A and S1B, respectively) and 9 differentially expressed microRNAs (5 up-regulated and 4 down-regulated microRNAs, see Additional file 1: Table S1C) from our previous genomic profiling studies on OTSCC [15, 16] for the development of this putative MRMs list. This putative MRMs list consists of 265 putative MRMs defined by microRNA up-regulation and mRNA down-regulation, and 176 putative MRMs defined by microRNA down-regulation and mRNA up-regulation. We then tested these putative MRMs using a panel of 12 different sequence-based microRNA target prediction algorithms (DIANAmT, miRanda, microCosm, miRDB, miRWalk, RNAhybrid, PicTar (4-way), PicTar (5-way), PITA, RNA22, TargetScan5.1, and TargetScanHuman6.2) to refine our putative MRMs list. A total of 132 candidate MRMs were identified (predicted as microRNA target by at least 1 bioinformatics algorithm, see Additional file 2: Table S2A and Additional file 3: Table S2B). As shown in Table 1, 38 potential MRMs were predicted by at least 3 bioinformatics target prediction algorithms, where the up-regulation of the microRNA contributes to the down-regulation of mRNA, and 15 potential MRMs were predicted by at least 3 bioinformatics target prediction algorithms (Table 2), where down-regulation of the microRNA contributes to the up-regulation of mRNA. The differential expression of microRNAs and coding genes (mRNAs) involved in these 53 potential MRMs (9 microRNAs and 34 mRNAs) was then validated using dataset on 12 OTSCC and paired normal tissues (extracted from TCGA Data Portal). As shown in Additional file 4: Table S3, statistically significant differential expression was observed for 8 out of 9 microRNAs and 23 out of 34 mRNAs tested in the validation OTSCC cohort.

**Table 1:** Putative microRNA-mRNA regulatory module defined by microRNA up-regulation and mRNA down-regulation^a^

Putative miR-mRNA regulatory module		Bioinformatics Prediction^c^	Correlation (TCGA dataset)^d^		Correlation (HNSCC cell line)^e^		Correlation (patient sample)^f^	
miR (up)^b^	mRNA (down)^b^		Pearson *r*	*p* value	Pearson *r*	*p* value	Pearson *r*	*p* value
hsa-miR-155	**ADH1B**	6	−0.3263	0.120034	−0.0317	0.914331		
**hsa**-**miR**-**31**	**ADH1B**	3	−0.3651	0.079472	0.3863			
**hsa**-**miR**-**223**	ADIPOQ	5	−0.3104	0.140425	0.476			
**hsa**-**miR**-**130b**	ADIPOQ	3	−0.3612	0.083075	0.2356			
**hsa**-**miR**-**223**	**ALOX12**	5	−0.2752	0.193414	0.5856			
**hsa**-**miR**-**130b**	ATP1A2	6	**−0.4324**	**0.035023**	−0.1899	0.515529		
**hsa**-**miR**-**31**	ATP1A2	3	−0.3265	0.120034	0.2494			
**hsa**-**miR**-**223**	CEACAM5	6	−0.0421	0.845504	−0.1689	0.563792		
**hsa**-**miR**-**21**	CEACAM5	5	−0.107	0.618738	−0.0834	0.776829		
**hsa**-**miR**-**130b**	CEACAM5	4	−0.1968	0.358677	−0.2497	0.389269		
**hsa**-**miR**-**223**	**CEACAM7**	6	−0.111	0.605605	−0.1364	0.641958		
**hsa**-**miR**-**21**	CILP	5	**−0.4095**	**0.047201**	−0.1815	0.534608		
**hsa**-**miR**-**21**	**CLU**	3	**−0.4126**	**0.045447**	−0.1612	0.581947		
**hsa**-**miR**-**31**	**EMP1**	5	−0.1491	0.487134	−0.0997	0.7345		
**hsa**-**miR**-**130b**	**EMP1**	4	**−0.5049**	**0.012034**	0.0951			
**hsa**-**miR**-**21**	**GPD1L**	5	**−0.6784**	**0.000269**	−0.4509	0.105628	**−0.9536**	**0.00001**
hsa-miR-155	**GPD1L**	5	−0.3008	0.154363	0.208			
**hsa**-**miR**-**223**	**HLF**	7	**−0.5536**	**0.005067**	0.0789			
**hsa**-**miR**-**31**	**HLF**	6	**−0.5107**	**0.010896**	0.2482			
**hsa**-**miR**-**130b**	**HLF**	6	**−0.62**	**0.001231**	0.067			
**hsa**-**miR**-**21**	**HLF**	3	**−0.7801**	**<0.00001**	**−0.5774**	**0.0307**	**−0.6707**	**0.048**
**hsa**-**miR**-**31**	**HPGD**	6	−0.2577	0.225391	0.0659			
**hsa**-**miR**-**21**	**HPGD**	6	**−0.55**	**0.005363**	**−0.5841**	**0.0283**	**−0.7972**	**0.0011**
**hsa**-**miR**-**130b**	**HPGD**	3	**−0.4602**	**0.023715**	−0.3617	0.203821		
**hsa**-**miR**-**21**	**ID4**	5	**−0.5229**	**0.008886**	−0.1121	0.702802		
**hsa**-**miR**-**31**	KRT15	3	−0.1438	0.505031	−0.0053	0.985653		
**hsa**-**miR**-**21**	LEPR	5	−0.2902	0.169251	0.6053			
**hsa**-**miR**-**223**	LEPR	4	−0.1443	0.502026	−0.3246	0.257504		
**hsa**-**miR**-**130b**	**MGLL**	4	**−0.6913**	**0.000183**	−0.5158	0.05903	−0.1864	0.542035
**hsa**-**miR**-**223**	**NEBL**	6	**−0.518**	**0.009519**	−0.0598	0.8391		
**hsa**-**miR**-**130b**	**NEBL**	5	**−0.5237**	**0.008733**	0.091			
**hsa**-**miR**-**21**	**NEBL**	3	**−0.5582**	**0.004605**	−0.1793	0.539655		
**hsa**-**miR**-**223**	**NMU**	5	−0.2119	0.322319	0.6125			
**hsa**-**miR**-**31**	**PPP1R3C**	4	−0.2695	0.203707	0.5588			
hsa-miR-155	**PTN**	6	0.1331		−0.1157	0.693673		
**hsa**-**miR**-**130b**	**TGM1**	7	**−0.5858**	**0.002676**	0.0162			
hsa-miR-155	**ZNF185**	6	−0.0065	0.977802	−0.2152	0.46		
**hsa**-**miR**-**21**	**ZNF185**	5	−0.3451	0.098725	−0.0739	0.8018		

^a^The putative microRNA-mRNA regulatory module (MRM) was constructed based on microRNA and mRNA expression profiles of OTSCC, as we previously reported in [16] and [15], respectively

^b^Differential expression of microRNAs and mRNAs was validated using dataset on 12 OTSCC and paired normal tissue samples that were extracted from TCGA. Genes that show statistically significant differential expression were identified with bold font

^c^The candidate targets of a microRNA were predicted using a collection of 12 bioinformatics tools, including DIANAmT, miRanda, microCosm, miRDB, miRWalk, RNAhybrid, PicTar (4-way), PicTar (5-way), PITA, RNA22, TargetScan5, and TargetScanHuman 6.2. The number of bioinformatics tools (out of a total of 12 tools tested here) that predict a gene to be a microRNA target was presented. The gene/microRNA pairs predicted by at least 3 tools were listed in the table

^d^Correlations of microRNA and mRNA levels were assessed using dataset on 12 OTSCC and paired normal controls that were extracted from TCGA. Inverted correlation (negative Pearson *r* value) is expected for a MRM, and p value was calculated

^e^Correlations of microRNA and mRNA levels were assessed by quantitative real-time PCR based on 13 HNSCC cell line. Inverted correlation (negative Pearson *r* value) is expected for a MRM, and *p* value was calculated

^f^Correlations of 4 pairs of microRNA and mRNA levels were assessed by quantitative real-time PCR based on 13 OTSCC patient oral cytology samples. Inverted correlation (negative Pearson r value) is expected for a MRM, and *p* value was calculated

**Table 2:** Putative microRNA-mRNA regulatory module defined by microRNA down-regulation and mRNA up-regulation^a^

Putative miR-mRNA regulatory module		Bioinformatics Prediction^c^	Correlation (TCGA dataset)^d^		Correlation (HNSCC cell line)^e^	
miR (down)^b^	mRNA (up)^b^		Pearson *r*	*p* value	Pearson *r*	*p* value
**hsa**-**miR**-**375**	**COL4A6**	5	−0.3145	0.13511	0.2432	
**hsa**-**miR**-**375**	**COL5A1**	5	−0.3659	0.079472	−0.2159	0.4585
**hsa**-**miR**-**125b**	**COL5A1**	5	**−0.437**	**0.03274**	−0.0325	0.9122
**hsa**-**miR**-**375**	**COL5A2**	5	−0.3708	0.075136	−0.231	0.426861
**hsa**-**miR**-**375**	CXCL1	4	−0.0864	0.689479	0.7146	
**hsa**-**miR**-**125b**	CXCL13	6	−0.3346	0.110688	−0.1736	0.552828
**hsa**-**miR**-**375**	**DFNA5**	4	**−0.4936**	**0.014374**	−0.0855	0.771344
**hsa**-**miR**-**100**	FSTL4	3	−0.0923	0.668975	−0.1796	0.538966
**hsa**-**miR**-**99a**	FSTL4	3	−0.1413	0.511067	−0.1847	0.527303
**hsa**-**miR**-**125b**	**HMGA2**	5	**−0.4628**	**0.023036**	−0.0557	0.849995
**hsa**-**miR**-**375**	IFI44L	4	−0.1937	0.366226	0.42	
**hsa**-**miR**-**125b**	**IGFBP3**	4	−0.3656	0.079472	−0.2774	0.336959
**hsa**-**miR**-**125b**	**LAMC2**	5	**−0.6952**	**0.000164**	0.3459	
**hsa**-**miR**-**375**	**LAMC2**	3	**−0.4309**	**0.035971**	0.4508	
**hsa**-**miR**-**375**	ODC1	3	−0.2375	0.264826	−0.1239	0.673024

^a^The putative microRNA-mRNA regulatory module (MRM) was constructed based on microRNA and mRNA expression profiles of OTSCC, as we previously reported in [16] and [15], respectively

^b^Differential expression of microRNAs and mRNAs was validated using dataset on 12 OTSCC and paired normal tissue samples that was extracted from TCGA. Genes that show statistically significant differential expression were identified with bold font

^c^The candidate targets of a microRNA were predicted using a collection of 12 bioinformatics tools, including DIANAmT, miRanda, microCosm, miRDB, miRWalk, RNAhybrid, PicTar (4-way), PicTar (5-way), PITA, RNA22, TargetScan5, and TargetScanHuman 6.2. The number of bioinformatics tools (out of a total of 12 tools tested here) that predict a gene to be a microRNA target was presented. The gene/microRNA pairs predicted by at least 3 tools were listed in the table

^d^Correlations of microRNA and mRNA levels were assessed using dataset on 12 paired OTSCC and normal controls that was extracted from TCGA Data Portal. Inverted correlation (negative Pearson *r* value) is expected for a MRM, and *p* value was calculated

^e^Correlations of microRNA and mRNA levels were assessed by quantitative real-time PCR based on 13 HNSCC cell line. Inverted correlation (negative Pearson *r* value) is expected for a MRM, and *p* value was calculated

**Fig. 1:**
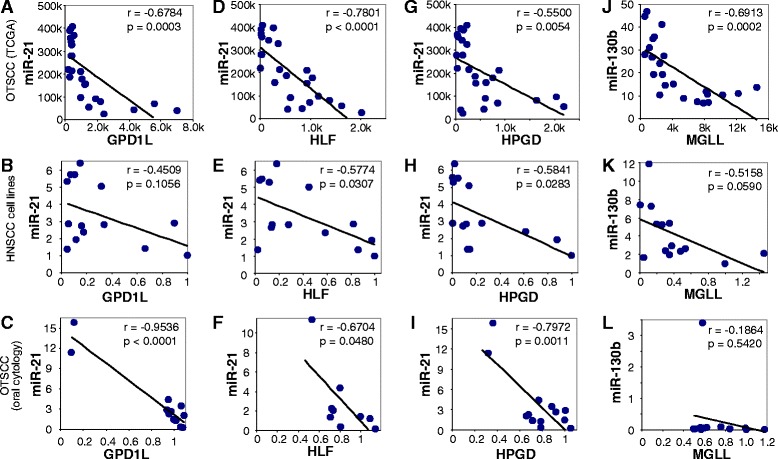
Correlation of microRNAs and the expression of their target genes in OTSCC. The levels of miR-21 and miR-130b and the expression of HPGD, GPD1L, HLF and MGLL were extracted for 12 OTSCC and paired normal tissue samples from The Cancer Genome Atlas (TCGA) (**a**, **d**, **g**, **j**), assessed by qRT-PCR on 13 HNSCC cell lines (**b**, **e**, **h**, **k**), and on 13 oral cytology samples from OTSCC patients (**c**, **f**, **i**, **l**). The correlation of the miR-21 level with the expression of GPD1L (**a**, **b**, **c**), HLF (**d**, **e**, **f**), HPGD (**g**, **h**, **i**), and the correlation of miR-130b level with the expression of MGLL (**j**, **k**, **l**) were assessed, and the Pearson’s correlation coefficient (r) was calculated

To further evaluate these potential MRMs, we examined the correlative relationship between the microRNA levels and the expression of their target genes in these 12 OTSCC and 12 paired normal tissues (extracted from TCGA Data Portal), as well as 13 HNSCC cell lines (Table 1 and Fig. 1). Among these 53 microRNA-mRNA pairs tested, 4 exhibited apparent inverse correlations in both OTSCC tissue samples and HNSCC cell lines (miR-21-GPD1L, miR-21-HLF, miR-21-HPGD and miR-130b-MGLL, with Pearson’s correlation coefficient *r* = −0.6784, −0.7801, −0.55, −0.6913 for OTSCC tissue samples, and *r* = −0.4509, −0.5774, −0.5841, and −0.5158 for HNSCC cell lines, respectively). The inverse correlations for miR-21-GPD1L, miR-21-HLF, miR-21-HPGD and miR-130b-MGLL were statistically significant in OTSCC tissue samples, and the inverse correlations for miR-21-HLF and miR-21-HPGD were also statistically significant in HNSCC cell lines.

We further evaluated these 4 MRMs in oral cytology samples from 13 OTSCC cases, and statistically significant inverse correlations were observed for miR-21-GPD1L, miR-21-HLF, and miR-21-HPGD, but not for miR-130b-MGLL (*r* = −0.9536, *p* = 0.00001; *r* = −0.6707, *p* = 0.048; *r* = −0.7972, *p* = 0.0011; and *r* = −0.1864, *p* = 0.542035; Table 1 and Fig. 1).

**Fig. 2:**
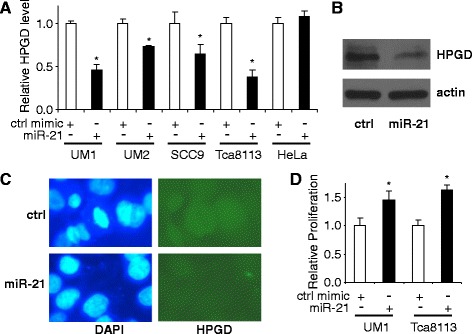
MiR-21-mediated down-regulation of HPGD and up-regulation of proliferation. **a** miR-21 mimic and negative control mimic were introduced into the UM1, UM2, SCC9 Tca8113 and HeLa cells. qRT-PCR was performed to assess the expression of HPGD. **b** Western blot was performed to assess the expression of HPGD at protein level in UM1 cells treated with either miR-21 mimic or negative control mimic. **c** The expression HPGD was measured by fluorescent immunocytochemical analysis in UM1 cells treated with either miR-21 mimic or negative control mimic (Green: HPGD; Blue: DAPI nuclear staining). **d** The UM1 and Tca8113 cells were treated with miR-21 mimic or negative control mimic, and the cell proliferation was assessed by MTT assay. Data represents at least 3 independent triplicate experiments with similar results. * indicates *p* < 0.05

We further explore the interaction of miR-21 and HPGD in our study. As shown in Fig. 2a, ectopic transfection of miR-21 mimic to UM1, UM2, SCC9 and Tca8113 cells led to a statistically significant reduction in HPGD mRNA level as compared to cells treated with control mimic. The miR-21 has no apparent effect on HPGD expression in HeLa cells. As shown in Fig. 2b and c, ectopic transfection of miR-21 mimic to UM1 cells led to reduced HPGD expression at protein level and reduced immunostaining of HPGD, respectively, as compared to the cells treated with control mimic. As shown in Fig. 2d, ectopic transfection of miR-21 mimic also enhanced the proliferation of OTSCC cells, which is consistent with previous observations [10, 32], and confirmed the oncogenic effect of miR-21.

**Fig. 3:**
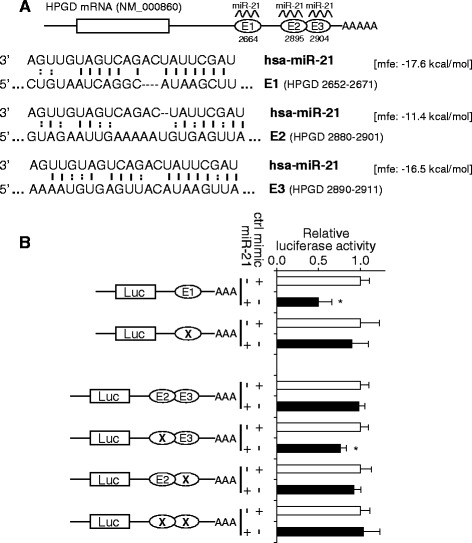
MiR-21 direct targeting HPGD mRNA. **a** Three predicted miR-21 targeting sites (E1, E2, E3) are located in the 3′-UTR of HPGD mRNA. The numbers under the diagram are the starting bp-position of the seed regions for the miR-21 targeting sites. The base-pairing and the minimum free energy (mfe) for the binding of miR-21 to the targeting sequences were predicted using the RNAhybrid program [18]. **b** Dual luciferase reporter assays were performed to test the interaction of miR-21 and its targeting sequences in the HPGD mRNA using constructs containing the predicted targeting sequences (pGL-E1 and pGL-E2E3) and mutated targeting sequences (pGL-E1m, pGL-E2mE3, pGL-E2E3m, pGL-E2mE3m) cloned into the 3′-UTR of the reporter gene. Data represent at least 3 independent experiments with similar results. *: *p* < 0.05

Bioinformatics analysis revealed that there are three miR-21 targeting sites located in the 3′-UTR of the HPGD mRNA (E1 at position 2652 to 2671, E2 at position 2880 to 2901, E3 at 2890 to 2911) and the targeting sites E2 and E3 are partially overlapped (Fig. 3a). The predicted minimum free energy (mfe) for the binding of these sites to miR-21 are −17.6, −11.4 and −16.5 kcal/mol, respectively. To test whether the miR-21 directly interacts with these predicted targeting sites in HPGD mRNA, dual luciferase reporter assays were performed using constructs containing these targeting sites (Fig. 3b). When cells were transfected with miR-21, the luciferase activities of the construct containing targeting site E1 (pGL-E1) was significantly reduced as compared to the cells transfected with negative control. When the seed region of this targeting site was mutated (pGL-E1m), the effect of miR-21 on the luciferase activity was abolished. For sites E2 and E3, when cells were transfected with miR-21, the luciferase activities of the construct containing both targeting sites E2 and E3 (pGL-E2E3) was not changes as compared to the cells transfected with negative control. Interestingly, when the seed region of E2 was mutated (pGL-E2mE3), the miR-21-mediated down-regulation of the luciferase activity was observed. MiR-21 has no effect on constructs with E3 mutation (pGL-E2E3m) or mutations of both E2 and E3 (pGL-E2mE3m).

**Fig. 4:**
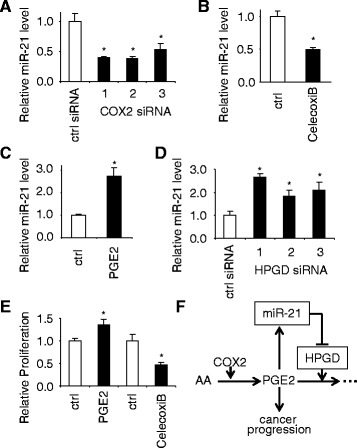
PGE2 regulates its own degradation by regulating miR-21 and its target gene HPGD. UM1 cells were treated with either control siRNA or specific siRNAs against COX2 (**a**), or treated with either COX2 inhibitor CelecoxiB or vehicle (**b**), or treated with either PGE2 or vehicle (**c**), or treated with either control siRNA or specific siRNAs against HPGD (**d**). The relative level of miR-21 was assessed by qRT-PCR. **e** UM1 cells were treated with PGE2, CelecoxiB or vehicle, and the cell proliferation was assessed by MTT assay. Data represent at least 3 independent experiments with similar results. *: *p* < 0.05. **f** Potential role of the positive feed-forward loop among PGE2, miR-21 and PHGD in OTSCC

As shown in Fig. 4a and b, both siRNA-mediated knockdown of COX2 and treatment with COX2 inhibitor (CelecoxiB) led to down-regulation of miR-21 in UM1 cells. As shown in Fig. 4c, directly apply PGE2 to the UM1 cells led to the up-regulation of miR-21, and knockdown of HPGD (Fig. 4d) also led to the up-regulation of miR-21. As anticipated, treating cells with PGE2 and CelecoxiB led to up-regulation and down-regulation of cell proliferation, respectively, which is consistent with previous observations [33, 34] (Fig. 4e). These results are in agreement with observation made by Lu et al. in cholangiocarcinoma [35], which confirm the PGE2-mediated miR-21 up-regulation in OTSCC and suggest a PGE2-miR-21-HPGD positive feed-forward loop that contributes to tumorigenesis (Fig. 4f).

## Discussion

Despite the significant increase in the number of experimentally validated microRNA-mRNA regulatory relationships, the majority of the microRNA targeted genes remains unknown. MicroRNA usually binds to its target genes through partial complementary. While numerous sequence-based bioinformatics methods for microRNA target prediction have been developed, these methods often lead to high false discovery rates [11]. However, the integration of these bioinformatics tools with mRNA/microRNA differential expression profiles often lead to the identification of high confidence microRNA-mRNA regulatory modules. In this study, we carried out this integrated analysis to identify MRMs in two steps. First, based on the simple principle that inverse relationships should be anticipated in the expression of a specific microRNA and its functional target genes, we developed a list of putative microRNA-mRNA regulatory modules by linking each microRNAs with all inversely regulated mRNAs based on the results of our previous mRNA and microRNA profiling studies on OTSCC [15, 16]. The second step is to these putative MRMs bioinformaticsly using sequence-based microRNA target prediction algorithm. Since there are many available sequence-based microRNA target prediction tools, and each of these tools utilizes a different model to define targeting sequences that are associated with functionality, the predictions differ when applied to the same microRNAs, with each method having different levels of coverage and false positive prediction [11]. In order to reduce the potential false positives, we used a voting scheme to combine the predictions from the 12 commonly used bioinformatics tools, including DIANAmT, miRanda, microCosm, miRDB, miRWalk, RNAhybrid, PicTar (4-way), PicTar (5-way), PITA, RNA22, TargetScan5.1, and TargetScanHuman6.2. With this integrated approach, we developed a list of 53 potential MRMs that are differentially expressed in OTSCC.

Since the microRNA regulates its target gene mainly at post-transcriptional level, inverse correlation between the levels of microRNA and mRNA pair is a key characteristic of a functional MRM. We further prioritized the list of differentially expressed MRMs in OTSCC by examining the correlative relationship between the microRNA levels and the expression of their target genes in 3 sets of samples (12 OTSCC and 12 paired normal tissues, 13 HNSCC cell lines, and 13 oral cytology samples from OTSCC cases). This comprehensive prioritization step led to 4 promising MRMs, including miR-21-GPD1L, miR-21-HLF, miR-21-HPGD and miR-130b-MGLL.

Deregulations of miR-21 and miR-130b, as well as deregulation of GPD1L, HLF, HPGD and MGLL have been reported either in HNOC or other cancer types [15, 16, 36–42], and these MRMs represent significant functional relevance in OTSCC. GPD1L has the glycerol-3-phosphate dehydrogenase enzyme activity and is a regulator of HIF-1α stability [40]. And a recent study showed that the GPD1L expression is a strong predictor for local recurrence and survival in HNSCC [39]. HLF belongs to the PAR (proline and acidic amino acid-rich) subfamily of bZIP transcription factors [43, 44], and plays a role in development and circadian rhythm regulation in the mammalian. HLF fusion proteins that resulted from chromosomal translocation (e.g., E2A-HLF) are often linked to leukemia. However, the role of HLF in OTSCC is not entirely clear. MGLL is involved in Prostaglandin E2 (PGE2) production in response to inflammation and infection which leads to fever [45]. Arachidonic acid (AA), a precursor for PGE2, is typically liberated from AA-containing phospholipids by the action of phospholipases A2 (PLA2s). MGLL is a monoacylglycerol lipase which hydrolyzes 2-arachidonoylglycerol (2-AG), an endocannabinoid that functions in the central nervous system, to AA and glycerol, representing an alternative AA-producing pathway. MGLL may also play a role in certain types of cancer by regulating both endocannabinoid and fatty acid pathways, and supporting protumorigenic metabolism [46]. This appears to be contradict with the apparent down-regulation of MGLL observed in OTSCC [15], and the miR-130b-MGLL regulatory module predicted here. Nonetheless, whether MGLL plays a role in OTSCC and, if so, by what mechanism are questions that remain unanswered. HPGD is a known anti-tumorigenic effecter, and it regulates the tumorigenic actions of Prostaglandin E2 (PGE2) by converts PGE2 to its biologically inactive metabolite, and down-regulation of HPGD has been observed in many human cancer types [47–53]. Since miR-21 is one of the most consistently observed up-regulated microRNA in OTSCC [16, 54], the miR-21-HPGD regulatory module may represents a critical mechanism of regulating PGE2 signaling.

Our functional study confirmed the effect of miR-21 on HPGD expression level, and the direct interaction of miR-21 with the HPGD mRNA in OTSCC cells. We identified three miR-21 targeting sites located in the 3′-UTR of the HPGD mRNA, including a previously reported site (E1) [35], and two partially overlapped sites (E2 and E3). While we confirmed the miR-21-mediated and E1 site-dependent target gene downregulation, E2 and E3 sites appear to have no effect. This may be because that targeting sites E2 and E3 are partially overlapped, and may interfere with the proper interaction with the RISC complex. The elimination of E2 may partially restore the capability of E3 (which has a stronger binding affinity among the two sites) to binding to the RISC complex. This is different than our previous observation where miR-138 was able to interact with multiple overlapping target sites on the FOSL1 mRNA [55]. Additional studies are needed to explore this mutual exclusive phenomenon among multiple targeting sites. The HPGD gene has 6 known transcript variants (NCBI accession: NM_000860, NM_001145816, NM_001256301, NM_001256305, NM_001256306, NM_001256307), and all 6 variants have the same 3′-UTR. As such, the interaction between miR-21 and HPGD mRNA is not likely to be affected by alternative splicing. Interestingly, we did not observe any miR-21 effect on HPGD expression in HeLa cells (a cell line that originated from a cervical cancer case). It is possible that this apparent difference in the miR-21 effect on HPGD expression may be due to differences in cancer types. It is worth noting that the effect of miR-21 on HPGD expression has also been observed in other cancer type [35]. Alternatively, this difference may be cell-line specific. HeLa cells (or the OTSCC cell lines used here) may have specific mutation(s) that dictate the miR-21 effects on HPGD. More in-depth functional analysis will be needed to fully evaluate the miR-21-HPGD regulatory module in different cancer types and in other biological systems.

The levels of COX2 and its catalytic product PGE2 are increased in a variety of malignancies, including HNOC [56–59]. The tumorigenic actions of PGE2 are attributable to its modulation of cell proliferation, survival, migration, and invasion. The level of PGE2 is controlled by the status of PGE2 synthesis and degradation. Whereas the cyclooxygenases (COX1 and COX2) are rate-limiting key enzymes that control PGE2 biosynthesis, HPGD is a key enzyme that converts PGE2 to its biologically inactive metabolite, 13,14-dihydro-15-keto-PGE2, thus leading to PGE2 inactivation [60, 61]. Consistent with the antitumorigenic effect of HPGD, the down-regulation of HPGD has been observed in many human cancer types [47–53]. Lu et al., first reported the PGE2-mediated up-regulation of miR-21 in cholangiocarcinoma, and suggested a positive feed-forward loop that involves PGE2, miR-21 and HPGD [35]. Our results are consistent with these previous observations, and confirm the existence of a PGE2-miR-21-HPGD positive feed-forward loop in OTSCC that contributes to tumorigenesis (Fig. 4f).

## Conclusions

In summary, we identified a number of high-confidence MRMs in OTSCC, including miR-21-GPD1L, miR-21-HLF, miR-21-HPGD and miR-130b-MGLL regulatory modules. Among these MRMs, miR-21-HPGD regulatory module may play an important role as part of a feed-forward loop that regulates the PGE2 signaling. Such a feed-forward regulatory mechanism likely plays a critical role in OTSCC initiation and progression. Thus, combining the COX2 inhibitor-based therapies with miR-21 inhibitors may represent a promising therapeutic strategy for treating patients with OTSCC.

## Additional files

Additional file 1: Table S1. Lists of 97 differentially expressed mRNA transcripts and 9 microRNAs in OTSCC based on our previous genomic profiling studies [15, 16]. (DOC 140 kb) Additional file 2: Table S2A. Candidate microRNA-mRNA regulatory module defined by microRNA up-regulation and mRNA down-regulation. (XLSX 15 kb) Additional file 3: Table S2B. Candidate microRNA-mRNA regulatory module defined by microRNA down-regulation and mRNA up-regulation. (XLSX 11 kb) Additional file 4: Table S3. Validation of the differential expression of microRNAs and mRNAs involved in putative MRM using TCGA dataset. (XLSX 12 kb)

## Abbreviations

HNOC: Head and neck/oral cancer
HNSCC: Head and neck squamous cell carcinoma
HPGD: 15-Hydroxyprostaglandin Dehydrogenase
mfe: Minimum free energy
MRM: microRNA-mRNA regulatory module
OTSCC: Oral tongue squamous cell carcinoma
TCGA: The cancer genome atlas
