# Supplementary material for: MicroRNA-21 regulates prostaglandin E2 signaling pathway by targeting 15-hydroxyprostaglandin dehydrogenase in tongue squamous cell carcinoma
Source: BMC Cancer. 2016 Aug 25;16(1):685. doi: 10.1186/s12885-016-2716-0 (PMC5000501; doi:10.1186/s12885-016-2716-0)
Supplement: Additional file 1: Table S1. — Lists of 97 differentially expressed mRNA transcripts and 9 microRNAs in OTSCC based on our previous genomic profiling studies [15, 16]. (DOC 140 kb) [file 12885_2016_2716_MOESM1_ESM.doc]

**Supplement Table S1A**: Up-regulated transcripts in OTSCC *

| **Probe ID** | **Gene Symbol** | **Gene Title** | **Chromosomal Location** | **p-value** | **Fold change** |
| --- | --- | --- | --- | --- | --- |
| 204475_at | MMP1 | matrix metallopeptidase 1 (interstitial collagenase) | 11q22.3 | 0 | 57.61528 |
| 205680_at | MMP10 | matrix metallopeptidase 10 (stromelysin 2) | 11q22.3 | 9.95E-06 | 8.448239 |
| 205828_at | MMP3 | matrix metallopeptidase 3 | 11q22.3 | 1.01E-08 | 8.434561 |
| 204580_at | MMP12 | matrix metallopeptidase 12 (macrophage elastase) | 11q22.3 | 7.39E-07 | 7.843088 |
| 211756_at | PTHLH | parathyroid hormone-like hormone | 12p12.1-p11.2 | 2.43E-07 | 7.567635 |
| 210511_s_at | INHBA | inhibin, beta A | 7p15-p13 | 2.95E-07 | 7.007183 |
| 202267_at | LAMC2 | laminin, gamma 2 | 1q25-q31 | 8.83E-08 | 6.874205 |
| 202859_x_at | IL8 | interleukin 8 | 4q13-q21 | 1.54E-06 | 5.866572 |
| 210809_s_at | POSTN | periostin, osteoblast specific factor | 13q13.3 | 0.000127 | 5.386487 |
| 205157_s_at | KRT17 | keratin 17 | 17q12-q21 | 1.70E-05 | 4.822128 |
| 202404_s_at | COL1A2 | collagen, type I, alpha 2 | 7q22.1 | 9.15E-07 | 4.683783 |
| 204415_at | IFI6 | interferon, alpha-inducible protein 6 | 1p35 | 1.24E-06 | 4.661726 |
| 205483_s_at | ISG15 | ISG15 ubiquitin-like modifier | 1p36.33 | 7.50E-06 | 4.615246 |
| 205479_s_at | PLAU | plasminogen activator, urokinase | 10q24 | 1.59E-08 | 4.364222 |
| 218468_s_at | GREM1 | gremlin 1, cysteine knot superfamily, homolog (Xenopus laevis) | 15q13-q15 | 3.66E-05 | 4.190351 |
| 203936_s_at | MMP9 | matrix metallopeptidase 9 | 20q11.2-q13.1 | 5.85E-05 | 4.079094 |
| 214453_s_at | IFI44 | interferon-induced protein 44 | 1p31.1 | 4.96E-07 | 4.057301 |
| 204470_at | CXCL1 | chemokine (C-X-C motif) ligand 1 (melanoma growth stimulating activity, alpha) | 4q21 | 1.44E-05 | 4.009033 |
| 37892_at | COL11A1 | collagen, type XI, alpha 1 | 1p21 | 0.000934 | 4.006326 |
| 221729_at | COL5A2 | collagen, type V, alpha 2 | 2q14-q32 | 5.60E-06 | 3.907361 |
| 211980_at | COL4A1 | collagen, type IV, alpha 1 | 13q34 | 2.62E-07 | 3.893552 |
| 205959_at | MMP13 | matrix metallopeptidase 13 (collagenase 3) | 11q22.3 | 0.000712 | 3.791227 |
| 213992_at | COL4A6 | collagen, type IV, alpha 6 | Xq22 | 1.99E-06 | 3.757882 |
| 205242_at | CXCL13 | chemokine (C-X-C motif) ligand 13 (B-cell chemoattractant) | 4q21 | 0.000218 | 3.695939 |
| 212364_at | MYO1B | myosin IB | 2q12-q34 | 8.29E-11 | 3.69239 |
| 203413_at | NELL2 | NEL-like 2 (chicken) | 12q13.11-q13.12 | 0.00026 | 3.546042 |
| 201506_at | TGFBI | transforming growth factor, beta-induced, 68kDa | 5q31 | 4.88E-05 | 3.519956 |
| 200790_at | ODC1 | ornithine decarboxylase 1 | 2p25 | 9.04E-05 | 3.500822 |
| 201645_at | TNC | tenascin C (hexabrachion) | 9q33 | 5.69E-05 | 3.493753 |
| 201641_at | BST2 | bone marrow stromal cell antigen 2 | 19p13.2 | 3.85E-06 | 3.425675 |
| 212473_s_at | MICAL2 | microtubule associated monoxygenase, calponin and LIM domain containing 2 | 11p15.3 | 2.14E-06 | 3.356392 |
| 203789_s_at | SEMA3C | sema domain, immunoglobulin domain (Ig), short basic domain, secreted, (semaphorin) 3C | 7q21-q31 | 7.58E-05 | 3.288134 |
| 202411_at | IFI27 | interferon, alpha-inducible protein 27 | 14q32 | 6.98E-05 | 3.276552 |
| 203256_at | CDH3 | cadherin 3, type 1, P-cadherin (placental) | 16q22.1 | 2.17E-07 | 3.264723 |
| 208025_s_at | HMGA2 | high mobility group AT-hook 2 | 12q15 | 0.0001 | 3.237605 |
| 209875_s_at | SPP1 | secreted phosphoprotein 1 | 4q21-q25 | 0.002693 | 3.234513 |
| 203510_at | MET | met proto-oncogene (hepatocyte growth factor receptor) | 7q31 | 2.17E-09 | 3.17755 |
| 202310_s_at | COL1A1 | collagen, type I, alpha 1 | 17q21.33 | 0.000451 | 3.169439 |
| 204948_s_at | FST | follistatin | 5q11.2 | 6.58E-06 | 3.168141 |
| 212488_at | COL5A1 | collagen, type V, alpha 1 | 9q34.2-q34.3 | 0.000308 | 3.06461 |
| 217901_at | DSG2 | Desmoglein 2 | 18q12.1 | 3.97E-05 | 3.049675 |
| 204320_at | COL11A1 | collagen, type XI, alpha 1 | 1p21 | 0.001018 | 3.049474 |
| 201852_x_at | COL3A1 | collagen, type III, alpha 1 | 2q31 | 0.000552 | 3.048682 |
| 203695_s_at | DFNA5 | deafness, autosomal dominant 5 | 7p15 | 2.58E-06 | 3.047308 |
| 210095_s_at | IGFBP3 | insulin-like growth factor binding protein 3 | 7p13-p12 | 3.42E-05 | 3.000012 |

* Based on and [Ye et al. 2008]. p value < 0.01; fold increase > 3.0.

Supplement Table S1B: Down-regulated transcripts in OTSCC *

| **Probe ID** | **Gene Symbol** | **Gene Title** | **Chromosomal Location** | **p-value** | **Fold change** |
| --- | --- | --- | --- | --- | --- |
| 213240_s_at | KRT4 | keratin 4 | 12q12-q13 | 1.67E-06 | 0.050358 |
| 204777_s_at | MAL | mal, T-cell differentiation protein | 2cen-q13 | 3.97E-07 | 0.0512 |
| 220090_at | CRNN | cornulin | 1q21 | 1.91E-06 | 0.053086 |
| 207935_s_at | KRT13 | keratin 13 | 17q12-q21.2 | 0.000509 | 0.08558 |
| 206884_s_at | SCEL | sciellin | 13q22 | 4.63E-07 | 0.110991 |
| 207802_at | CRISP3 | cysteine-rich secretory protein 3 | 6p12.3 | 6.51E-05 | 0.120375 |
| 218990_s_at | SPRR3 | small proline-rich protein 3 | 1q21-q22 | 0.002721 | 0.135765 |
| 205185_at | SPINK5 | serine peptidase inhibitor, Kazal type 5 | 5q32 | 7.68E-06 | 0.142829 |
| 220026_at | CLCA4 | chloride channel, calcium activated, family member 4 | 1p31-p22 | 6.75E-06 | 0.152554 |
| 209612_s_at | ADH1B | alcohol dehydrogenase IB (class I), beta polypeptide | 4q21-q23 | 1.59E-08 | 0.15979 |
| 206605_at | P11 | 26 serine protease | 12q13.1 | 9.62E-08 | 0.163667 |
| 206004_at | TGM3 | transglutaminase 3 | 20q11.2 | 1.61E-05 | 0.172884 |
| 219554_at | RHCG | Rh family, C glycoprotein | 15q25 | 9.96E-05 | 0.190647 |
| 204284_at | PPP1R3C | protein phosphatase 1, regulatory (inhibitor) subunit 3C | 10q23-q24 | 8.37E-06 | 0.208755 |
| 206199_at | CEACAM7 | carcinoembryonic antigen-related cell adhesion molecule 7 | 19q13.2 | 5.59E-06 | 0.214557 |
| 203914_x_at | HPGD | hydroxyprostaglandin dehydrogenase 15-(NAD) | 4q34-q35 | 9.16E-08 | 0.226633 |
| 209687_at | CXCL12 | chemokine (C-X-C motif) ligand 12 (stromal cell-derived factor 1) | 10q11.1 | 0.000785 | 0.228046 |
| 205382_s_at | CFD | complement factor D (adipsin) | 19p13.3 | 1.40E-06 | 0.228892 |
| 213371_at | LDB3 | LIM domain binding 3 | 10q22.3-q23.2 | 0.001125 | 0.230323 |
| 204719_at | ABCA8 | ATP-binding cassette, sub-family A (ABC1), member 8 | 17q24 | 5.16E-06 | 0.231521 |
| 222043_at | CLU | clusterin | 8p21-p12 | 1.06E-05 | 0.239177 |
| 214235_at | CYP3A5 | cytochrome P450, family 3, subfamily A, polypeptide 5 | 7q21.1 | 4.63E-07 | 0.246038 |
| 211737_x_at | PTN | pleiotrophin (heparin binding growth factor 8, neurite growth-promoting factor 1) | 7q33-q34 | 4.96E-07 | 0.251359 |
| 209763_at | CHRDL1 | chordin-like 1 | Xq22.3 | 2.31E-05 | 0.25263 |
| 211597_s_at | HOP | homeodomain-only protein | 4q11-q12 | 0.000109 | 0.262894 |
| 201325_s_at | EMP1 | epithelial membrane protein 1 | 12p12.3 | 5.37E-07 | 0.266213 |
| 201884_at | CEACAM5 | carcinoembryonic antigen-related cell adhesion molecule 5 | 19q13.1-q13.2 | 0.000769 | 0.270884 |
| 214063_s_at | TF | Transferring | 3q22.1 | 0.000785 | 0.271463 |
| 212510_at | GPD1L | glycerol-3-phosphate dehydrogenase 1-like | 3p22.3 | 7.43E-08 | 0.280631 |
| 203961_at | NEBL | Nebulette | 10p12 | 1.81E-06 | 0.284048 |
| 214399_s_at | KRT8 | Keratin 8 | 12q13 | 5.59E-06 | 0.29015 |
| 210096_at | CYP4B1 | cytochrome P450, family 4, subfamily B, polypeptide 1 | 1p34-p12 | 4.20E-05 | 0.294115 |
| 204570_at | COX7A1 | cytochrome c oxidase subunit VIIa polypeptide 1 | 19q13.1 | 0.000342 | 0.300063 |
| 220431_at | TMPRSS11E | transmembrane protease, serine 11E | 4q13.2 | 0.000281 | 0.303501 |
| 204753_s_at | HLF | hepatic leukemia factor | 17q22 | 2.15E-07 | 0.304885 |
| 209365_s_at | ECM1 | extracellular matrix protein 1 | 1q21 | 8.24E-06 | 0.30796 |
| 207761_s_at | METTL7A | methyltransferase like 7A | 12q13.13 | 0.000162 | 0.308639 |
| 204483_at | ENO3 | enolase 3 (beta, muscle) | 17pter-p11 | 0.006533 | 0.312551 |
| 206023_at | NMU | neuromedin U | 4q12 | 6.31E-05 | 0.314672 |
| 209242_at | PEG3 | paternally expressed 3 | 19q13.4 | 0.000818 | 0.320181 |
| 215704_at | FLG | Filaggrin | 1q21.3 | 0.00116 | 0.321361 |
| 211026_s_at | MGLL | monoglyceride lipase | 3q21.3 | 3.80E-07 | 0.321579 |
| 207175_at | ADIPOQ | adiponectin, C1Q and collagen domain containing | 3q27 | 0.00035 | 0.322687 |
| 206008_at | TGM1 | transglutaminase 1 (K polypeptide epidermal type I, protein-glutamine-gamma-glutamyltransferase) | 14q11.2 | 0.003627 | 0.322892 |
| 203296_s_at | ATP1A2 | ATPase, Na+/K+ transporting, alpha 2 (+) polypeptide | 1q21-q23 | 0.006235 | 0.323235 |
| 207602_at | TMPRSS11D | transmembrane protease, serine 11D | 4q13.2 | 0.002622 | 0.324075 |
| 213451_x_at | TNXB | tenascin XB | 6p21.3 | 1.32E-07 | 0.324392 |
| 203585_at | ZNF185 | zinc finger protein 185 (LIM domain) | Xq28 | 6.93E-05 | 0.325255 |
| 207206_s_at | ALOX12 | arachidonate 12-lipoxygenase | 17p13.1 | 5.88E-05 | 0.325564 |
| 206227_at | CILP | cartilage intermediate layer protein, nucleotide pyrophosphohydrolase | 15q22 | 2.08E-05 | 0.326899 |
| 209894_at | LEPR | leptin receptor | 1p31 | 0.000161 | 0.32778 |
| 209291_at | ID4 | inhibitor of DNA binding 4, dominant negative helix-loop-helix protein | 6p22-p21 | 7.00E-08 | 0.327922 |
| 201348_at | GPX3 | glutathione peroxidase 3 (plasma) | 5q23 | 0.000175 | 0.330296 |

* Based on and [Ye et al. 2008]. p value < 0.01; fold change < 0.333

**Supplement Table S1C: Differentially expressed microRNAs in OTSCC ***

| **MicroRNA** | **Chromosomal location** | **Mature miR sequence** | **p-value** | **Fold change** |
| --- | --- | --- | --- | --- |
| miR-21 | 17q23.1 | uagcuuaucagacugauguuga | 0.006 | 8.15572 |
| miR-155 | 21q21.3 | uuaaugcuaaucgugauaggggu | 0.004 | 6.61830 |
| miR-130b | 22 | cagugcaaugaugaaagggcau | 0.039 | 15.5948 |
| miR-223 | Xq12 | ugucaguuugucaaauacccca | 0.002 | 3.13698 |
| miR-31 | 9p21.3 | aggcaagaugcuggcauagcu | 0.002 | 9.44345 |
|  |  |  |  |  |
| miR-100 | 11q24.1 | aacccguagauccgaacuugug | 0.002 | 0.49841 |
| miR-99a | 21q21.1 | aacccguagauccgaucuugug | 0.002 | 0.57168 |
| miR-375 | 2q35 | uuuguucguucggcucgcguga | 0.002 | 0.37502 |
| miR-125b | 11q24.1 or 21q21.1 | ucccugagacccuaacuuguga | 0.064 | 0.57983 |

* MicroRNA differential expression was consistently reported by at least 4 out of 13 studies used in meta-analysis described in [Chen et al. 2012], and experimentally confirmed in paired normal and OTSCC tissue samples.
